# Transcriptome Analysis Reveals the Molecular Mechanism of *Pseudomonas* with Different Adhesion Abilities on *Tilapia* Decay

**DOI:** 10.3390/foods14050795

**Published:** 2025-02-26

**Authors:** Liumin Zhuang, Chen Song, Yunru Wei, Jinzhi Han, Li Ni, Chengxu Ruan, Wen Zhang

**Affiliations:** Institute of Food Science and Technology, College of Biological Science and Engineering, Fuzhou University, Fuzhou 350108, China; 230820076@fzu.edu.cn (L.Z.); m13277453709@163.com (C.S.); 18960918975@163.com (Y.W.); hjz419@fzu.edu.cn (J.H.); nili@fzu.edu.cn (L.N.)

**Keywords:** *Pseudomonas*, adhesion genes, NaCl regulation, spoilage

## Abstract

This study aimed to investigate the molecular mechanism of *Pseudomonas* with varying adhesion capabilities to *Tilapia*’s intestinal mucus influence the spoilage potential of *Tilapia*. Sodium chloride(NaCl) was used as an environmental factor to regulate *Pseudomonas*’ adhesion ability. After being exposed to 3.5% NaCl stress, the PS01 strain with low adhesion showed an enhancement in adhesion ability, while the LP-3 strain with high adhesion exhibited a decrease. Correspondingly, the expression of critical adhesion genes, such as *flgC*, *fliC*, and *cheB*, was found to be altered. LP-3, with high adhesion ability, was observed to promote a relative increase in *Nocardioides* and *Cloacibacterium* in fish intestines. This led to the production of more volatile compounds, including 2-octen-1-ol Z, 2,3-Octanedione, and Eicosane, thus deepening the spoilage of tilapia. LP-3, with reduced adhesion ability after NaCl regulation, showed a diminished capacity to cause fish spoilage. Transcriptomics analysis was used to examine two *Pseudomonas* strains that exhibited different adhesion abilities, leading to the identification of an adhesion regulatory network involving flagellar assembly regulation, bacterial chemotaxis, quorum sensing, two-component systems, biofilm formation, and bacterial secretion systems. This study identified the *Pseudomonas* adhesion regulatory pathway and determined 10 key adhesion-related genes.

## 1. Introduction

*Pseudomonas* spp. is commonly found in the mucus or intestinal linings of fish [1,2,3] and is known to contribute to the process of fish spoilage [4]. Among the various bacterial strains that are commonly researched, *Pseudomonas fluorescens* and *Pseudomonas putida* are recognized as the primary culprits of fish spoilage [5,6,7]. However, it is worth noting that variations in adhesion and spoilage capabilities do exist among different strains of these bacteria [8,9].

Adhesion serves as the initial step in the formation of biofilms [10]. When bacteria reach the surface of fish mucus, the interaction between the bacterial cell surface and the fish mucus surface leads to the formation of a biofilm. Therefore, the ability to form biofilms indirectly reflects the adhesion capacity of the bacteria [11]. This complex process of interaction between bacteria and host cells can be divided into two stages: the non-specific binding stage and the specific adhesion stage, where the bacteria bind to specific receptors on the body surface [12].

The adhesion of *Pseudomonas* is predominantly associated with flagella, adhesins, and biofilm formation. Currently, few studies have been conducted to investigate the internal mechanisms of adhesion at the gene level. Estrella [13] screened 67 adhesion-related genes that are shared by *Pseudomonas putida* and *Pseudomonas aeruginosa*. However, despite extensive research on the role of *Pseudomonas* spp. in fish spoilage [14,15,16], several gaps remain. The genetic mechanisms underlying the adhesion of *Pseudomonas* spp. are not fully understood, particularly in relation to environmental factors such as NaCl concentration. Additionally, the direct link between *Pseudomonas* adhesion ability and its influence on fish spoilage remains unclear.

From this perspective, the objective of this study is to identify the key genes that play a pivotal role in *Pseudomonas fluorescens* and *Pseudomonas putida* adhesion and explore the impact of NaCl as an environmental factor on *Pseudomonas* adhesion. Moreover, we aim to investigate the impact of *Pseudomonas* adhesion on their colonization and spoilage of fish. This study conducted transcriptomic analysis on two *Pseudomonas* strains with varying adhesion capabilities and identified genes that were differentially expressed but co-expressed. Furthermore, in vitro measurements were taken of the adhesion rates of two *Pseudomonas* strains with high and low adhesion after being treated with NaCl. Moreover, high-throughput sequencing was used to analyze changes in the intestinal microbiota and volatile compounds in spoiled fish and to establish a correlation between microbial genera and volatile compounds. The findings of this study are intended to provide a theoretical reference not just for the understanding of Pseudomonas adhesion and its role in fish spoilage but also for the future development of fish vaccines targeting the adhesion molecules of *Pseudomonas* spp.

## 2. Materials and Methods

### 2.1. Experimental Materials, Strains

The bacterial strains employed in this investigation were obtained through isolation and purification from spoiled tilapia, which was identified as *Pseudomonas putida* LP-3 (GenBank accession number OL597895) and *Pseudomonas fluorescens* PS01 (GenBank accession number OL597893).

The two *Pseudomonas* strains were inoculated in Luria-Bertani(LB)broth (Biosharp, Beijing, China) and incubated at 30 °C overnight, centrifuged using a low-temperature Centrifuge CF16RX (Hitachi, Ltd., Tokyo, Japan) at 8000 rpm for 10 min and then washed twice with phosphate buffer saline(PBS). Finally, resuspended with PBS to a concentration of approximately 1 × 10^8^ CFU/mL (OD600 = 1.0 ± 0.05).

To regulate the *Pseudomonas*, a final concentration of 3.5% NaCl was added to the LB broth before the inoculation, resulting in 3.5% NaCl-regulated *Pseudomonas*. The same approach was used to prepare a bacterial suspension with a concentration of approximately 1.0 × 10^8^ CFU/mL (OD_600_ = 1.0 ± 0.05).

Live *Tilapia* (*Oreochromis mossambicus*) was generously donated by the Fuzhou Aquaculture Research Institute and was temporarily raised in the pool for 30 days before the commencement of the experiment.

### 2.2. Determination of Adhesion Ability and Flagellar Observation

The present study involved the preparation of *Tilapia* intestinal mucus, which was carried out using the Larsen method [17]. Subsequently, *Pseudomonas* strains were fluorescently labeled and refined in accordance with the technique expounded by Vinderola [18]. The *Pseudomonas* strains were cultured at 30 °C for 18 h, after which they were centrifuged at 8000 rpm for 10 min at 4 °C and washed twice with PBS. A bacterial suspension was then prepared in 0.1 mol/L carbonate buffer, pH 9.2, which was sterilized and adjusted to a concentration of approximately 1.0 × 10^8^ CFU/mL. Fluorescein Isothiocyanate (FITC) was added to this bacterial solution at a final concentration of 0.2 mg/mL, and the cells were incubated in the dark at 30 °C for 1.5 h at 100 rpm. The bacterial suspension was subsequently washed several times with PBS until the solution became colorless. The suspension was then prepared again to a concentration of 1.0 × 10^8^ CFU/mL stored in the dark at 4 °C and used within 2 h.

The in vitro adhesion test was conducted using Zhang’s method [9,19] with some modifications. Specifically, 150 μL of the prepared mucus was added to each well of a 96-well microplate, which was coated overnight (fixed for 24 h) at 4 °C and washed twice with 200 μL sterile PBS. Subsequently, 150 μL of the FITC bacterial solution was added to each well and incubated at 30 °C for 1.5 h. Non-adherent *Pseudomonas* was removed by washing twice with sterile saline, and 150 μL of 1% (*w*/*w*) Sodium dodecyl sulfate(SDS) was added to lyse the bacteria at 60 °C for 1 h. Finally, the microplate was placed in a Multifunctional Microplate Reader SpectraMaxi3+MiniMax (Molecular Devices, Shanghai, China) for detection, with three replicates per group. A blank control with no mucus was used for comparison.

The adhesion rate was calculated using Equation (1).

Adhesion rate (%) = (E − C)/(F − B) × 100%(1)

where E = fluorescence intensity of experimental group, C = control group, F = pure FITC, and B = fluorescence intensity of bacterial solution.

In the meantime, we used transmission electron microscopy (TEM) to clearly observe the changes in flagella. Absorb 10 μL of bacterial solution on the copper mesh, stand still, and use a small piece of filter paper to absorb the excess liquid at the edge of the copper mesh. Negative staining was performed using a 1.5% phosphotungstic acid solution, and finally, the morphology of the flagella was visualized using a HT7700 transmission electron microscope (Hitachi, Ltd., Tokyo, Japan).

### 2.3. Sample Collection

**Figure 1:**
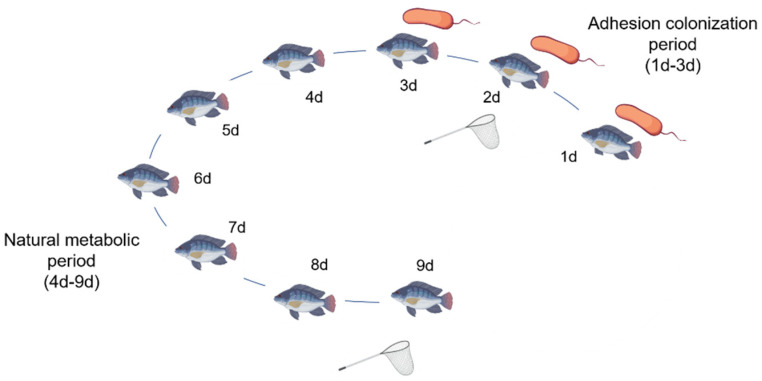
Animal testing procedure. The initial 1–3 day period was designated as the adhesion and colonization stage, while the subsequent 4–9 day period was classified as the natural metabolic stage. Fish samples were collected on both the 2nd and 9th day, respectively.

Two hundred tilapias, with an average body weight of 20 g, were randomly divided into five groups following a period of temporary culture for 30 days. The control group was fed normal feed in a water environment without the addition of bacterial liquid. The other four groups constituted the experimental groups, with each group receiving 5 mL of LP-3 bacterial solution (1.0 × 10^8^ CFU/mL), PS01 bacterial solution (1.0 × 10^8^ CFU/mL), 3.5% NaCl-regulated LP-3 bacterial solution (1.0 × 10^8^ CFU/mL), and 3.5% NaCl-regulated PS01 bacterial solution (1.0 × 10^8^ CFU/mL), respectively, from day 1 to day 3. The fish within the same group were placed in two separate plastic buckets (20 fish per bucket, 20 L capacity). As showed in Figure 1, Days 1 to 3 marked the period of adhesion and colonization, with approximately 50% to 70% daily water changes. Each morning, 5 mL of bacterial suspension was added. Days 4 to 9 were considered the natural metabolic period, with daily water changes.

On days 2 and 9, fish samples were collected and cleaned, and 200 mg of intestinal contents were extracted and placed in a 2 mL centrifuge tube for DNA extraction and microbial sequencing. Furthermore, the remaining fish bodies were washed with sterile water, placed in a sterile homogenization bag, and briefly stored at −20 °C for around 20 min to rapidly cool their temperature to simulate the real process. They were then transferred to a 4 °C refrigerator for three days, and 2.0 ± 0.1 g fish fillets were cut right before the determination of the volatile compounds.

### 2.4. DNA Extraction of Intestinal Microbiota, High-Throughput Sequencing Analysis, and qPCR Reaction

The extraction of fish intestinal genomic DNA was achieved utilizing a DNA extraction kit. Moreover, the V3-V4 region of the bacterial 16S rRNA gene was amplified using bacterial primers 341-F (5′-CCTAYGGGRBGCASCAG-3′) and 806-R (5′-GGACTACNNGGGTATCTAAT-3′). To facilitate testing, analysis, and plotting, the samples were sent to Shanghai Meiji Biomedical Technology Co., Ltd. and subjected to the Meiji Bioinformatics Cloud platform.

Furthermore, gene-specific primers were designed as follows [9]: *Pseudomonas* colonies were detected via qPCR, utilizing

Pse-F (5′-CTGCATCATGGCCTGACACATTT-3′) and Pse-R (5′-GTCGCATGGCTCGGTCTTCAGATC-3′).

The total number of bacteria was detected through qPCR using bacterial primers 27-F (5′-AGAGTTTGATCCTGGCTCAG-3′) and 1492-R (5′-GGTTACCTTGTTACGACTT-3′).

### 2.5. Volatile Components of Tilapia Meat

The method published by Zhang et al. [20] was used to determine volatile flavor compounds with appropriate modifications. After the sample collection, 2 g of fish sample was homogenized with 18 mL of sterile saline solution for 2 min. Then, the homogenized sample was transferred into a 15 mL extraction vial. Following this, 5 mL of saturated NaCl solution was added. To determine the absolute concentration of volatile components, 10 μL of 2-octanol with an initial concentration of 10 mg/L was added to the fish samples. The bottle was immediately sealed before being placed in a water bath for 3 min at 60 °C.

A pretreated SPME fiber (2.0 cm, 50/30 µm Divinylbenzene/Carboxen^®^/Polydimethylsiloxane, Supelco, Bellefonte, PA, USA) was then inserted into the vial for headspace adsorption for 30 min. Subsequently, the fiber head was introduced into the GC-MS (7890-B/5977 A, Agilent Technologies, Palo Alto, CA, USA) inlet, desorbed for 3 min and then removed. An HP-INNOWax capillary column (30 m × 0.32 mm × 0.15 μm) was used for detection. Helium as a carrier gas was maintained at 1 mL/min flow. The GC oven started at 40 °C, held at that temperature for 3 min and then ramped up to 120 °C at a 5 °C/min rate, then the temperature was increased to 230 °C at a 20 °C/min rate and finally held at that temperature for 5 min. The ion source and quadrupole temperatures were maintained at 200 °C and 150 °C, respectively.

Absolute concentration was then obtained by calculating the ratio of the peak area of the volatiles to 2-octanol (assuming the absolute correction factor of each volatile was 1.0) using the subsequent equation:

V (μg/g) = [(Ai/A) × 0.1]/m(2)

where V = Volatile component concentration, Ai = the peak area of volatile component i, A = the peak area of 2-Octanol, 0.1 = the amount of 2-Octanol added (μg), and m = the mass of the sample (g). It is assumed that the peak area of any peak has a linear relationship with concentration, and it is assumed that the peak area of the peak represents the same amount of material as the peak area of the standard 2-octanol.

### 2.6. RNA Extraction, cDNA Synthesis and Transcriptome Sequencing

LP-3 and PF01 were incubated in LB medium with the supplement of an additional 3.5% NaCl for 18 h and then centrifuged at 8000 rpm for 10 min at 4 °C and finally resuspended into a bacterial suspension with PBS. Total RNA was extracted from both strains using an RNA kit in accordance with the manufacturer’s guidelines. The extracted RNA was then treated with DNaseI to remove DNA. Following this, reverse transcription was immediately performed into cDNA, utilizing a reverse transcription kit on ice in accordance with the manufacturer’s instructions.

The reverse transcription process was synthesized into cDNA through a PCR reaction. The task of transcriptome sequencing was delegated to Shanghai Meiji Biomedical Technology Co., Ltd, Shanghai, China.

### 2.7. Enrichment Analysis and Differentially Expressed Genes

The genome and transcripts of the reference strain *Pseudomonas* sp. R32 (GenBank ID: 1573704) were aligned.

To create gene sets for genes with significant differential expressions in the LP-3 group and PS01 group, GO and KEGG functional enrichment analyses were conducted on the gene sets.

To analyze differential gene expressions between the LP-3 group and PS01 group, the Read Counts number of genes/transcripts was obtained. Subsequently, DESeq2 3.11 software based on the negative binomial distribution was employed to statistically analyze the raw counts [21], with the default parameter set as *p*-adjust < 0.05.

### 2.8. Validation of Adhesion Key Genes mRNA Expression After NaCl Regulation by RT-qPCR

For RNA-Seq verification purposes, 10 adhesion key genes (*yajC*, *flgC*, *flgG*, *flgH*, *flgI*, *fliC*, *fliA*, *livK*, *cheB*, *secY*) were analyzed, with 16S rRNA serving as the internal reference primer. The StepOnePlus Real-Time PCR System was utilized to configure the qPCR system on ice, following the qPCR kit guidelines and a Real-Time PCR reaction was performed.

The amplification conditions comprised pre-denaturation at 95 °C for 30 s, denaturation at 95 °C for 5 s, annealing at 55 °C for 30 s, and extension at 72 °C for 30 s, for a total of 42 cycles. The melting curve analysis program was set as follows: 95 °C for 15 s, 60 °C for 30 s, and 95 °C for 15 s.

Following the reaction, the Ct value was obtained, and the fold change of gene expression was calculated utilizing the 2^−ΔΔCt^ method [22].

### 2.9. Statistical Analysis

All measurements underwent independent repetition three times. The results were conveyed as mean ± standard deviation and depicted using GraphPad Prism 9.0. Two-way ANOVA analysis was executed, with significance established as *p* < 0.05.

To visually represent the data better, a PCoA (Principal Coordinates Analysis) was specifically applied to compare microbial community structures among different groups and a PCA (Principal Component Analysis) biplot was utilized to analyze and visualize the volatile compound profiles in fish samples across different groups. PCoA and PCA were generated utilizing SIMCA14.1. The connection between functional microbiota and volatile compounds was explicated through OPLS modeling. Additionally, a network analysis diagram was fashioned using Cytoscape 3.9.1.

## 3. Results

### 3.1. The Adhesion Difference of Pseudomonas

The adhesion rate of *Pseudomonas putida* LP-3 was over 30%, while that of *Pseudomonas fluorescens* was only slightly more than 10%. In vitro observations indicated significant differences in the adhesion of these two *Pseudomonas* strains. NaCl concentration (*w*/*v*) is a prevalent and extensively researched environmental factor. Previous studies have identified notable distinctions in spoilage and pathogenic bacteria in freshwater and marine fish. Ibryamova’s study shows that the species Pseudomonas is characteristic of marine Teleostei and is important for the life and metabolism of these vertebrates [23]. At the same time, *Pseudomonas fluorescens* are considered opportunistic pathogens that occur naturally in the aquatic environment and in the gut microbiota of healthy fish [24].

The spoilage microbiota of fish is composed almost exclusively of *Pseudomonas* spp. and *Shewanella putrefaciens* when it is under aerobic iced storage, whereas Gram-positive bacteria are likely spoilers of CO_2_-packed fish from fresh or tropical waters [6].

**Figure 2:**
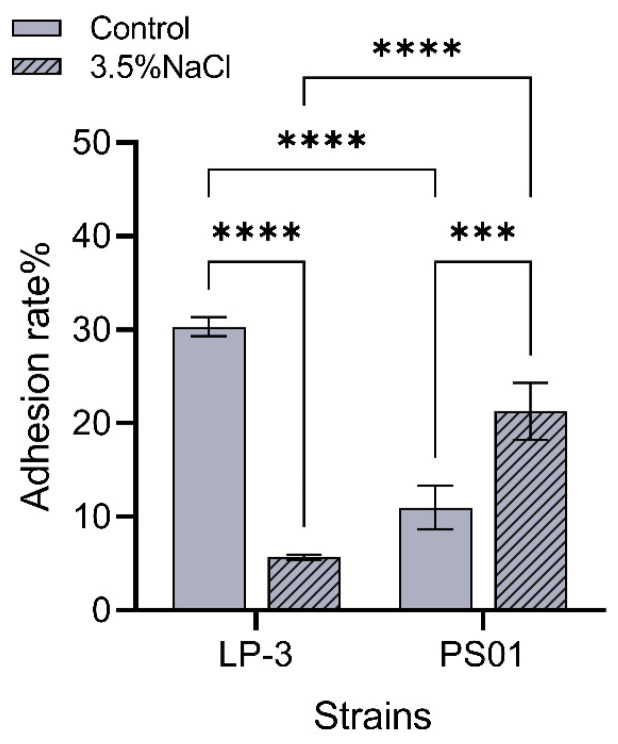
Adhesion difference of two Pseudomonas strains in vitro. Note: The value bars with ns are not significant; the asterisk denotes statistically significant differences; *** *p* < 0.001 and **** *p* < 0.0001.

To further investigate whether NaCl regulates *Pseudomonas* adhesion, the adhesion performance of the strains was evaluated under seawater-like conditions (mass fraction of NaCl about 3.5%). The results are depicted in Figure 2. Upon reaching an NaCl concentration of 3.5%, the adhesion ability of the two *Pseudomonas* strains exhibited different patterns. Specifically, the adhesion ability of *Pseudomonas fluorescens* PS01 increased with the increasing NaCl concentration, with the adhesion rate surpassing 20%. In contrast, the adhesion ability of *Pseudomonas putida* LP-3 decreased to less than 10% as the NaCl concentration increased.

**Figure 3:**
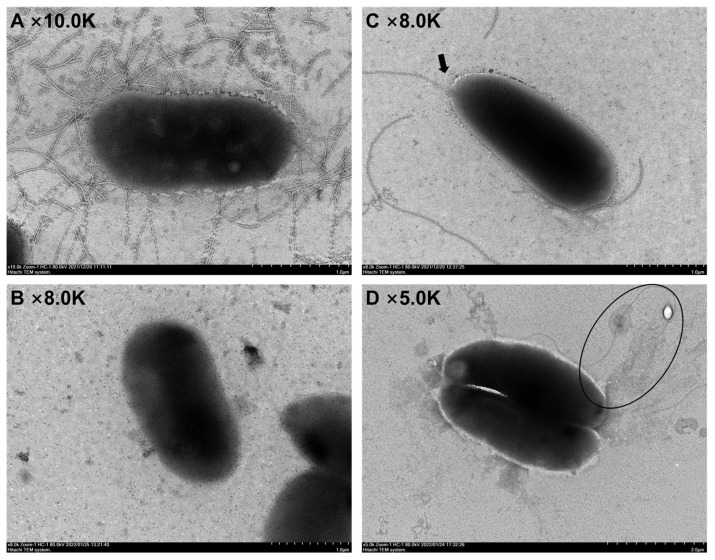
(**A**) TEM observation of LP-3, (**B**) TEM observation of PS01, (**C**) TEM observation of LP-3 after 3.5% NaCl treatment, (**D**) TEM observation of PS01 after 3.5% NaCl treatment.

The surface morphology of the strains was meticulously observed through transmission electron microscopy, as demonstrated in Figure 3. Notably, 3.5% NaCl regulation significantly altered the surface morphology of *Pseudomonas putida* LP-3 and *Pseudomonas fluorescens* PS01.

Before regulation, the flagella structure of *Pseudomonas putida* LP-3 was abundant, as depicted in Figure 3A. However, after regulation, the flagella structure surrounding Figure 3C disappeared. In comparison, *Pseudomonas fluorescens* PS01 had only a few flagella before regulation, as seen in Figure 3B, while after regulation in Figure 3D, the flagella were slender and had bent and aggregated in shape.

By comparing the before and after images, we hypothesize that the difference in the number and structure of flagella may be one of the underlying reasons for the difference in adhesion ability between LP-3 and PS01 strains. Regulation with 3.5% NaCl affects the adhesion of *Pseudomonas* by influencing the rotation and synthesis of flagella.

### 3.2. The Colonization of Pseudomonas in Tilapia Intestine Was Determined by qPCR and High Throughput

**Figure 4:**
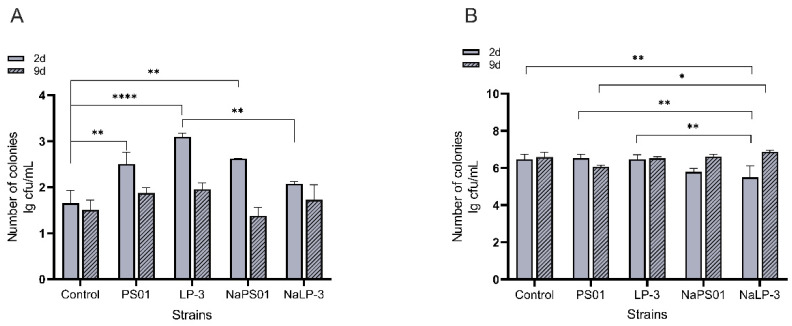
qPCR analysis of bacterial growth in fish intestine. (**A**) Number of intestinal *Pseudomonas* colonies measured by qPCR, (**B**) Number of total intestinal bacterial colonies measured by qPCR. Note: Not significant is not marked on the figure; 2d and 9d data do not compare; the asterisk denotes statistically significant differences * *p* < 0.05; ** *p* < 0.01; and **** *p* < 0.0001, 2d means the second day is the adhesion colonization period, 9d means the ninth day is the natural metabolic period; NaLP_3 is LP-3 strain regulated by 3.5% NaCl, NaPS01 is PS01 strain regulated by 3.5% NaCl.

The colonization of *Pseudomonas* strains with varying adhesion capabilities in the tilapia intestine was thoroughly evaluated using qPCR and high-throughput sequencing techniques. The adhesion and colonization of *Pseudomonas*, as well as the total number of bacteria in the fish intestine on the 2nd and 9th days, were quantitatively determined through qPCR (Figure 4).

On the 2nd day, *Pseudomonas* adhesion and colonization. The data clearly showed that, except for the NaLP-3 group, the number of *Pseudomonas* colonies in other experimental groups was substantially higher than that in the control group. The results indicated that the colonization ability of the LP-3 strain decreased significantly following 3.5% NaCl regulation (*p* < 0.01). However, no significant difference was observed in the colonization ability of the PS01 strain before and after regulation.

On the 9th day, the natural metabolic period occurred, and *Pseudomonas* in each group were excreted from the intestine. There was no significant difference in the number of *Pseudomonas* colonies in each group. Interestingly, there was no significant difference in the total bacterial content of the intestinal tract between the two periods. However, strains regulated with 3.5% NaCl during the adhesion colonization period showed a significant decrease in total bacterial colonization (*p* < 0.01). Overall, especially after the natural metabolic period, the adhesion and colonization of *Pseudomonas* to the intestinal tract did not significantly affect the total number of bacteria.

*Pseudomonas* adhesion and colonization in the intestine do not have a significant impact on the total number of microbiota colonies, but they can certainly affect the composition of the fish intestinal microbiota.

The PCoA was specifically applied to compare microbial community structures among different groups based on their abundance profiles. This method was suitable for analyzing our 16S rRNA sequencing data, as it effectively handles the ecological distance matrices derived from microbial community composition.

**Figure 5:**
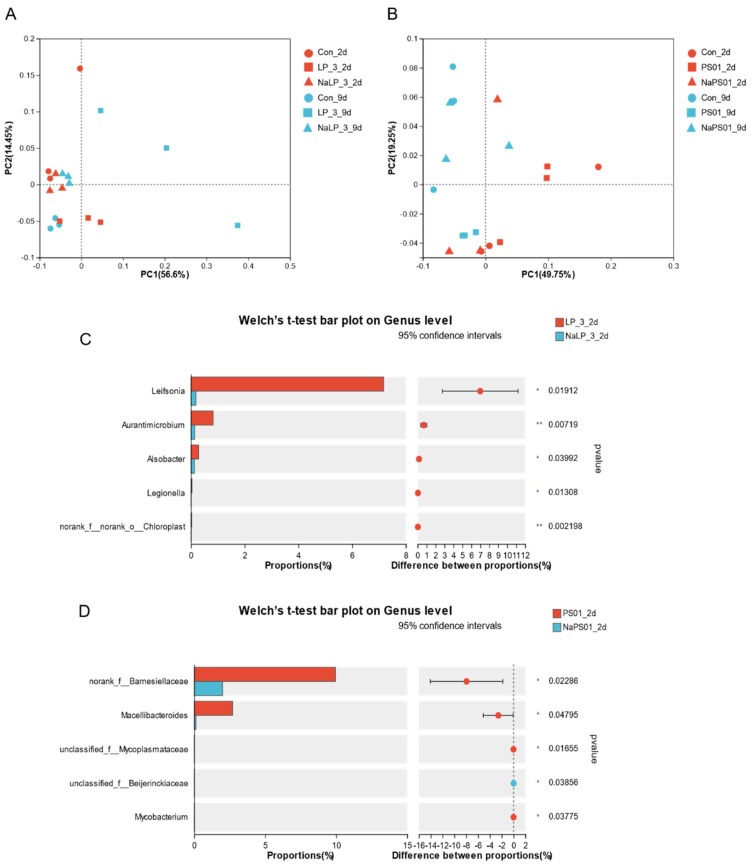
Comparative analysis of species diversity between groups and Species composition of intestinal microbial communities. (**A**) PCoA Analysis Chart of LP-3 Treatment Group, (**B**) PCoA Analysis Chart of PS01 Treatment Group, (**C**) Analysis of the difference between LP_3_2d group and NaLP_3_2d group, (**D**) Analysis of the difference between PS01_2d group and NaPS01_2d group; 2d means the second day is the adhesion colonization period, 9d means the ninth day is the natural metabolic period; NaLP_3 is LP-3 strain regulated by 3.5% NaCl, NaPS01 is PS01 strain regulated by 3.5% NaCl.

As shown in Figure 5A, the samples were clearly grouped into three regions: the LP_3_2d group, the LP_3_9d group, and the other groups (Con_2d, NaLP_3_2d, NaLP_3_9d). The LP-3 group showed significant differences before and after natural metabolism (2d and 9d), suggesting that the addition of the high adhesion strain LP-3 had a significant impact on reshaping the intestinal microbiota. In contrast, the NaLP_3_2d and NaLP_3_9d groups were more closely clustered with the early blank group, indicating that the adhesion ability of LP-3 was reduced by NaCl regulation without significantly altering the microbiota.

In Figure 5B, the PS01 group could be differentiated before and after natural metabolism (2d and 9d), despite the relatively small difference, suggesting that the addition of the low adhesion strain PS01 did have an effect on the intestinal tract, but it was less significant than the high adhesion strain LP-3. Although Figure 5B is less distinct than Figure 5A, it still showed the trend of distinguishing the PS01_2d group from the NaPS01_2d group and the PS01_9d group. The NaPS01_2d group itself did not cluster well, indicating that the introduction of PS01, which improved its adhesion ability after NaCl regulation, disrupted the intestinal microbiota. However, after the natural metabolic period (NaPS01_9d), it became more similar to the blank group on the 9th day, suggesting that the effect of NaCl on the adhesion ability of PS01 was limited, and its impact on the intestine was reduced by natural metabolism.

Based on the distinct clustering patterns revealed by the PCoA analysis, we performed comprehensive statistical analysis to identify differentially abundant species among the microbial communities of the LP_3_2d group, NaLP_3_2d group, PS01_2d group, and NaPS01_2d group, as illustrated in Figure 5C,D.

Upon meticulous analysis of the NaLP_3_2d group, we made a noteworthy observation of a significant decrease in the abundance of *Leifsonia*, *Aurantimicrobium*, and *Alsobacter*. However, we did not detect any apparent differential dominant bacteria. This intriguing result suggests that NaCl treatment reduced the adhesion of LP-3, consequently decreasing its impact on the microbiota.

On the other hand, in the case of the NaPS01_2d group, we made a compelling observation of a significant decrease in the abundance of *Barnesisllaceae* and *Macellibacteroides*, while the abundance of *Beijerinckiaceae* increased significantly [25,26]. This finding gives us a better understanding of the effect of NaCl on the adhesion and colonization of *Pseudomonas* with different adhesion abilities in the intestine of tilapia.

### 3.3. Effect of Pseudomonas on Fish Flavor

Specific spoilage organisms (SSO), which are microorganisms known to cause spoilage [27], have been associated with fish spoilage due to their ability to produce volatile compounds. These compounds can serve as an indicator of spoilage and can be used to examine the relationship between *Pseudomonas* adhesion and its spoilage ability before and after regulation.

During the adhesion colonization period on day 2 and the natural metabolic period on day 9, twenty-five volatile compounds were detected and meticulously screened using SPME-GC-MS. These compounds, including alcohols, aldehydes, esters, and aromatic compounds, were identified as potentially related to the presence of *Pseudomonas*.

PCA was employed to analyze and visualize the profiles of volatile compounds in fish samples across different experimental groups. This multivariate statistical technique was selected for its capacity to effectively reduce the dimensionality of our GC-MS data while retaining the variance in volatile organic compounds, thereby facilitating the identification of key compounds that contribute to the differentiation among groups.

**Figure 6:**
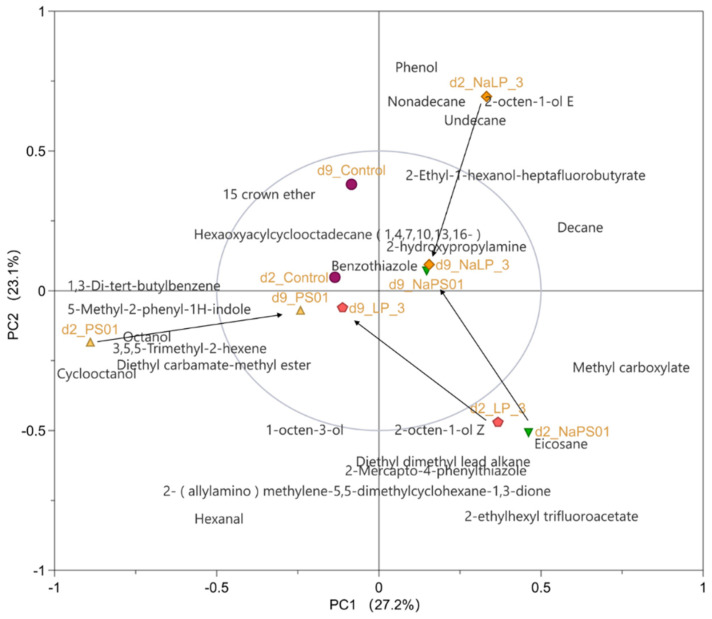
PCA Biplot of volatile compounds in different treatment groups. The middle circle is represented by a 50% Confidence Ellipse. Note: 2d means the second day is the adhesion colonization period, 9d means the ninth day is the natural metabolic period; NaLP_3 is the LP-3 strain regulated by 3.5% NaCl, and NaPS01 is the PS01 strain regulated by 3.5% NaCl.

The biplot based on the PCA-X model analysis, presented in Figure 6, revealed several intriguing findings. Firstly, during the natural metabolic period on day 9, both the experimental group and the control group were concentrated in the middle region (within the 50% confidence circle). Secondly, the LP-3 strain with high adhesion and the PS01 strain with improved adhesion after regulation, gathered and exhibited similar volatile compounds, such as 2-octen-1-ol Z, 2-Mercapto-4-phenylthiazole, Eicosane, and others. Thirdly, after regulation, the adhesion of LP-3 decreased and was situated in the first quadrant, exhibiting volatile compounds such as 2-octen-1-ol E and others. Lastly, the PS01 strain, which had low adhesion, was situated in the third quadrant and showed volatile compounds such as 3,5,5-Trimethyl-2-hexene and others.

**Figure 7:**
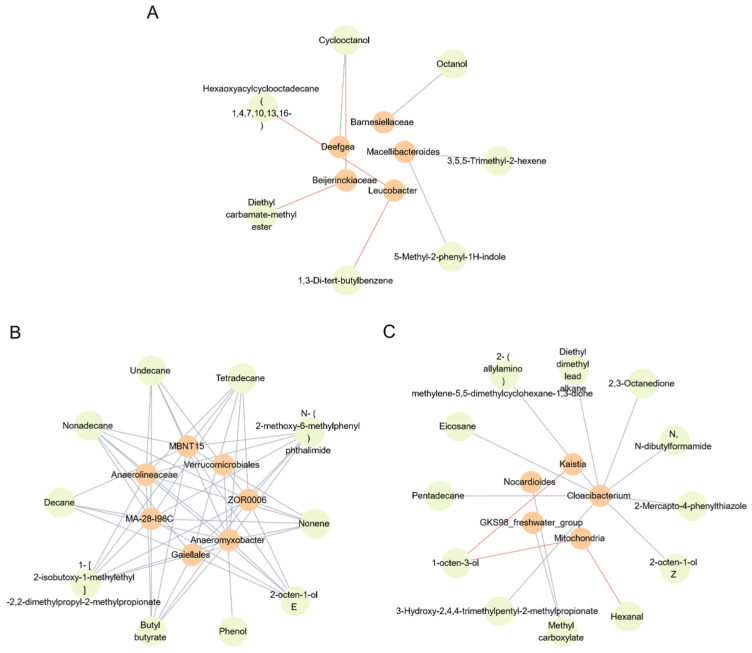
Network analysis of intestinal microbiota and volatile flavor compounds. Note: Negative correlation (light orange solid line). Positive correlation (ice blue solid line). (**A**) Correlations between intestinal microbiota and volatile flavor compounds of tilapias after feeding the PS01 strain, (**B**) Correlations between intestinal microbiota and volatile flavor compounds of tilapias after feeding the NaLP-3 strain, (**C**) Correlations between intestinal microbiota and volatile flavor compounds of tilapias after feeding the LP-3 and NaPS01 groups. NaLP_3 is the LP-3 strain regulated by 3.5% NaCl, and NaPS01 is the PS01 strain regulated by 3.5% NaCl.

Spearman’s correlation analysis was performed on the top 50 species at the genus level abundance and their associated volatile compounds to explore the intricate relationship between microbial genera and volatile compounds. A subset of data with a Spearman’s correlation coefficient of |R| > 0.7 (29 volatile compounds and 17 microbial genera) was meticulously selected. The resulting correlation network model was created using Cytospace 3.9.1software, as depicted in Figure 7 In this figure, the light orange node represents the microbial genus, while the light green node represents the volatile compounds. Negative correlations are indicated by light orange solid lines, and positive correlations are represented by ice blue solid lines.

As illustrated in Figure 6 and Figure 7, our findings revealed that feeding the PS01 strain caused significant flavor changes due to the increase of several genera (Figure 7A). Specifically, in the low-adhesion PS01 group, an increase in the genus *Macellibacteroides*, a marker for evaluating the freshness and deterioration of fish [28,29,30,31], led to a notable increase in the volatile compound 3,5,5-Trimethyl-2-hexene.

Similarly, feeding the NaCl-regulated LP-3 strain resulted in the emergence of distinct flavor changes caused by the increase of several strains (Figure 7B). In the NaLP_3 group, we detected a significant change in the diversity of the genus, but only 2-octen-1-ol E could be used as a marker of fish spoilage [32]. After feeding the LP-3 strain or NaCl-regulated PS01 strain, the flavor changes caused by the increase of several genera were observed (Figure 7C).

In the LP_3 group and NaPS01 group with high adhesion, we discovered that the proportion of *Nocardioides* and *Cloacibacterium* increased. Additionally, the proportion of bacteria belonging to the freshwater bacterial group GKS98 also increased [33]. This led to a significant increase in several volatile compounds (Figure 7C), including 2-octen-1-ol Z, a marker of moderate toxigenic strains (MT) [34]; 2,3-Octanedione, a marker of lipid oxidation-related spoilage [35,36]; Pentadecane and 2-Mercapto-4-phenylthiazole, which are accumulated in fish due to enzymatic or non-enzymatic lipid oxidation [28,37]; and Eicosane, which may affect the adhesion and biofilm formation of strains [38].

### 3.4. KEGG and GO Enrichment Analysis of Key Adhesion Gene Sets

**Figure 8:**
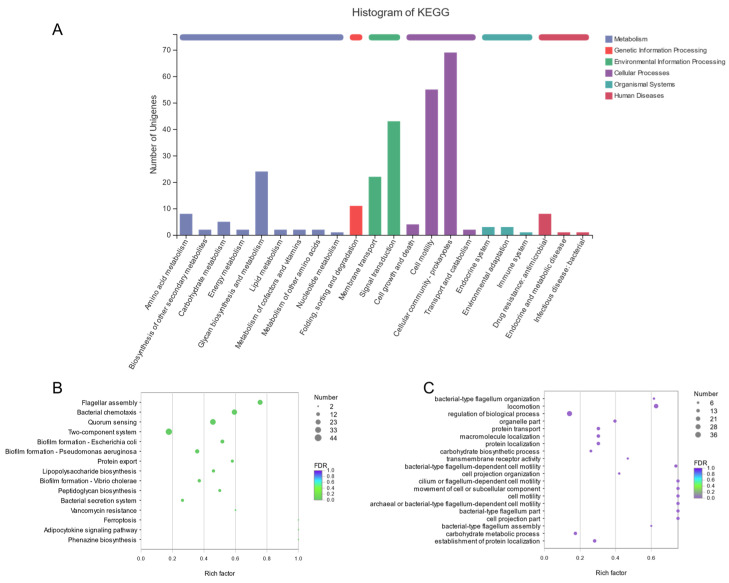
KEGG and GO enrichment analysis of K_adhesion gene sets. (**A**) KEGG Pathway Statistics of K_adhesion, (**B**) KEGG enrichment analysis of K_adhesion, (**C**) GO enrichment analysis of K_adhesion.

To explore the intricate molecular mechanisms underlying adhesion in greater depth, we meticulously selected adhesion-related genes, including bacterial adhesion, biofilm formation, bacterial motility, glucose metabolism, and membrane transport, among the differential genes. These genes were used to generate a gene set named K_adhesion. Subsequently, we selected a portion of the gene set with a co-expression of more than 10 to generate a refined gene set named coexpression10. The sophisticated KEGG pathway classification statistics of the K_adhesion gene set, presented in Figure 8A, revealed that the highest proportion of genes was associated with cell processes, where the prokaryotic cell community and cell movement were the main contributors. Notably, differences in the cell movement pathway were considered adhesion-related differential genes. Additionally, membrane transport, signal transduction, and carbohydrate metabolism also accounted for a significant portion of the environmental information process.

Further analysis of the KEGG functional enrichment maps, presented in Figure 8B,C, revealed a strong focus on flagellar formation, bacterial chemotaxis, quorum sensing, two-component systems, and biofilm formation. Similarly, the GO functional enrichment primarily focused on the regulation of biological processes, movement, and protein localization. These two sophisticated methods of gene function enrichment further refined the function of adhesion-related gene enrichment, providing a more comprehensive understanding of the molecular pathways involved in adhesion.

### 3.5. Screening of Key Adhesion Genes

**Figure 9:**
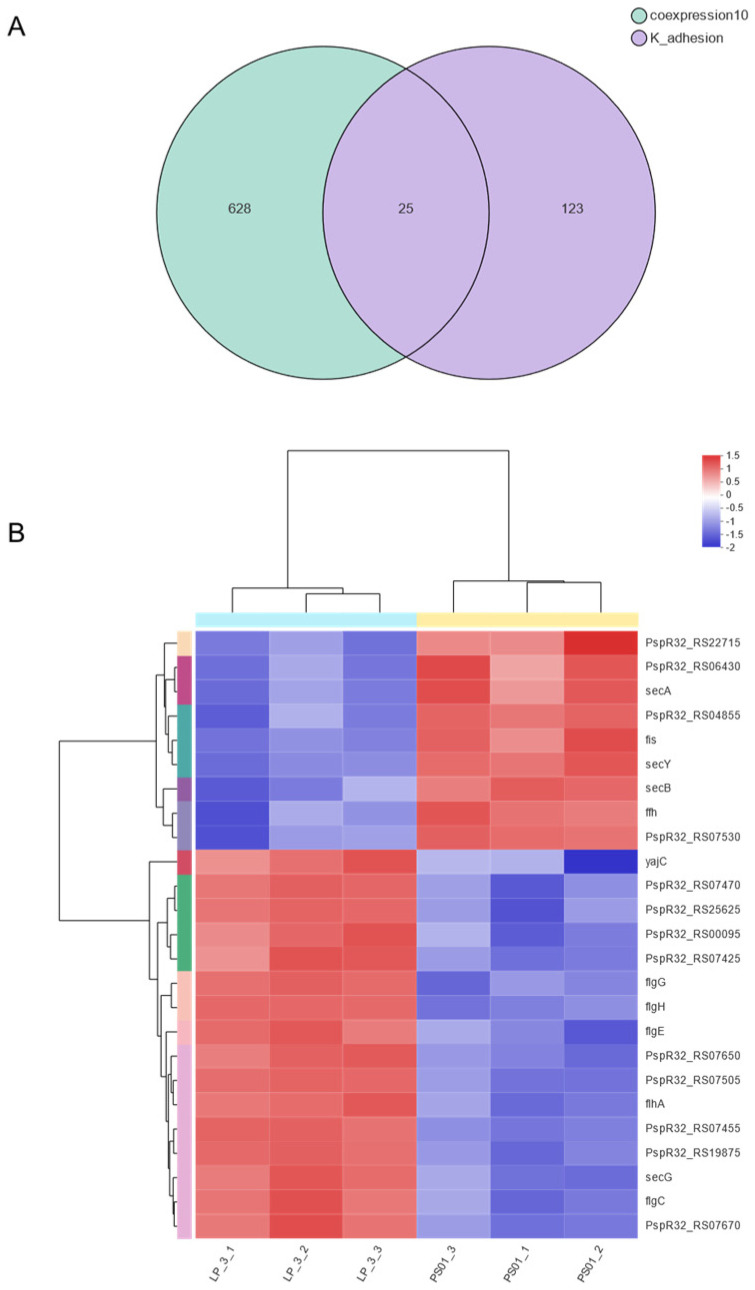
Gene set analysis. (**A**) Venn analysis, (**B**) The heatmap of 25 differentially expressed genes.

In our quest to unravel the molecular mechanisms underlying the difference in adhesion ability between *P. putida* LP-3 and *P. fluorescens* PS01, we employed Venn analysis on the co-expression gene set (green) and adhesion-related gene set (purple) of both strains (Figure 9A). This meticulous analysis identified 25 overlapping genes(Figure 9B), which were selected as the target key genes for further investigation. From this set of genes, we carefully handpicked 10 differentially expressed genes with a fold change of three times and christened them as key adhesion genes.

Remarkably, showed in Table 1, the expression levels of these key genes were significantly higher in *P. putida* LP-3 (high adhesion) than in *P. fluorescens* PS01 (low adhesion). Intriguingly, most of these key genes were flagellin-related genes, accompanied by some chemotactic proteins, sigma factors, and transmembrane protein-related genes. These novel findings suggest that the difference in flagella is the primary cause of the difference in adhesion between the two *Pseudomonas* strains, and there is a certain association with some membrane surface-related pathways. These remarkable insights provide a more profound understanding of the intricate molecular pathways involved in adhesion and could have significant implications for developing innovative strategies to control bacterial adhesion.

### 3.6. RT-qPCR Analysis of mRNA Expression of Key Genes for Adhesion Before and After Regulation

**Figure 10:**
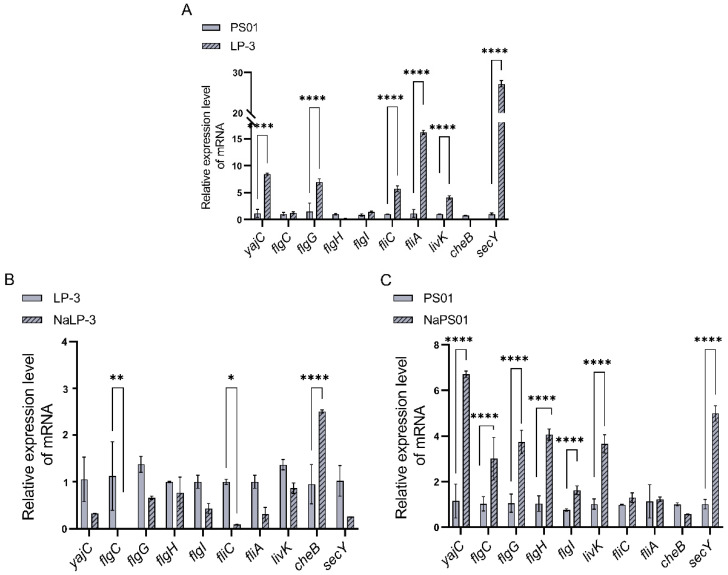
Expression of key adhesion genes. (**A**) Expression of key adhesion genes in LP -3 and PS01, (**B**) Expression of key adhesion genes in LP-3 after NaCl regulation, (**C**) Expression of key adhesion genes in PS01 after NaCl regulation. Note: The value bars with ns are not significant; the asterisk denotes statistically significant differences * *p* < 0.05; ** *p* < 0.01and **** *p* < 0.0001, NaLP-3 is LP-3 strain regulated by 3.5% NaCl, NaPS01 is PS01 strain regulated by 3.5% NaCl.

To verify the differential expression of 10 key adhesion genes in PS01 and LP-3, we employed RT-qPCR. The comparison results are presented in Figure 10A. Notably, five genes related to flagellar assembly (*flgG*, *fliC*, *fliA*) and bacterial secretion system (*livK*, *secY*) exhibited significantly higher expression in LP-3 than in PS01.

Our findings suggest that regulation with 3.5% NaCl significantly altered the adhesion ability of *Pseudomonas putida* LP-3 and *Pseudomonas fluorescens* PS01, primarily through the modulation of gene expression. Specifically, the adhesion ability of *Pseudomonas putida* LP-3 decreased significantly after 3.5% NaCl regulation, which mainly regulated the expression of *flgC* and *fliC*. In contrast, the adhesion ability of *Pseudomonas fluorescens* PS01 was enhanced after 3.5% NaCl regulation, primarily through the significant expression of *yajC*, *flgC*, *flgG*, *flgH*, *livK*, and *secY*.

These findings are consistent with previous reports that 3.5% NaCl can influence the adhesion ability of *Pseudomonas putida* strains both in vitro and in vivo. Our results suggest that NaCl, as an external environmental factor, can regulate the expression of key adhesion genes, leading to changes in the adhesion ability of these strains.

## 4. Discussion

The adhesion and colonization of *Pseudomonas* in the intestine can have a significant impact on the diversity of the intestinal microbiota. Recent studies have shown that the composition of the intestinal microbiota in fish is closely linked to fish spoilage [2,9,39,40].

In this study, we studied the effect of the adhesion ability of *Pseudomonas* on fish spoilage by high-throughput sequencing and detection of volatile substances in fish. We also used transcriptomics technology to identify genes and metabolic pathways that contribute to differences in adhesion ability.

Our results demonstrated that the addition of *Pseudomonas putida* LP-3, which exhibits high adhesion ability, and *Pseudomonas fluorescens* PS01, which displays enhanced adhesion ability after 3.5% NaCl regulation, resulted in more spoilage and pathogenic bacteria in the intestine compared to other strains. These findings suggest that these strains have a more detrimental effect on the flavor of fish.

PS01, which exhibits low adhesion ability, can induce an increase in *Barnesiellaceae*, *Macellibacteroides*, and *Deefgea* and a decrease in *Beijerinckiaceae* and *Leucobacter* in the intestine. Notably, *Macellibacteroides* has emerged as the dominant genus in this group and is known to produce volatile odors, such as 3,5,5-Trimethyl-2-hexene, which is a marker of fish deterioration.

Meanwhile, *Pseudomonas putida* LP-3, with its high adhesion ability, and *Pseudomonas fluorescens* PS01, with enhanced adhesion ability after 3.5% NaCl regulation, can induce an increase in *Nocardioides*, *Cloacibacterium*, and other bacteria in the intestine. *Nocardioides* are typically involved in the inflammatory process in animals [41,42] and can be isolated from soil and wastewater [43,44]. *Cloacibacterium* has been shown to increase rapidly during the cold storage of sea bream [45] and during inflammatory reactions in the intestinal mucosa [46], indicating its crucial role. As we previously mentioned, volatile flavors, such as 2-octen-1-ol Z, 2,3-Octenadione, Pentadecane, 2-Mercapto-4-phenylthiazole, and Eicosane, were detected in fish, significantly contributing to fish spoilage. Furthermore, we found that the *Leucobacter* genus, which diminishes in response to PS01 with low adhesion ability, becomes the dominant genus in response to LP-3. Previous studies have reported that the *Leucobacter* genus appears during the chilled European bass process and causes continuous spoilage for up to 12 days [47].

Despite a decrease in adhesion ability, LP-3 regulated by 3.5% NaCl can still induce an increase in *Anaeromyxobacter*, *Verrucomicrobiales*, *Anaerolineaceae*, *Gaiellales*, and other bacteria in the intestine. Compared to the direct addition of LP-3 with high adhesion ability, although the flavor became more diverse, only the volatile 2-octen-1-ol E had a greater impact on the corruption of fish meat.

These differences in flavor are intimately linked to the adhesion ability of *Pseudomonas*, with NaCl playing a regulatory role as an environmental factor. The high adhesion strain LP-3 and the PS01 strain with increased adhesion ability after 3.5% NaCl regulation induce an increase in pathogenic or spoilage bacteria. Compared to the PS01 strain itself, we detected more volatile compounds associated with spoilage.

Transcriptome analysis revealed that differences in adhesion between the two *Pseudomonas* species may be attributed to 10 genes. QPCR results confirmed that the genes with the most significant differences in adhesion between the two *Pseudomonas* were *fliA*, *fliC*, *flgG*, *livK*, *secY*, and others. Ultimately, the study determined that flagellar assembly regulation and bacterial secretion systems play vital regulatory roles in *Pseudomonas* adhesion.

In *Pseudomonas*, flagellar assembly regulation is highly conserved with 59 related genes [48]. A three-level regulatory system is typically used to explain the transcription of flagellar genes in *Pseudomonas putida* based on the time of their expression and their order of participation in regulation.

At the top of flagellar transcriptional regulation are genes such as *rpoN* and *fleQ*, which play different roles in regulating flagellar gene expression. RpoN encodes a sigma54 factor (RpoN) that binds to the promoter region, recruiting RNA polymerase to initiate transcription [49,50]. On the other hand, *fleQ* encodes a transcriptional enhancer, FleQ, that binds to the upstream region of the promoter, enhancing gene expression [51,52,53].

The second-level genes are activated by FleQ, which initiates the assembly of the flagellar basal body. Among them, *flgG*, together with *flgC*, *flgH*, *flgI*, *flgF*, and *flgE*, is essential for the composition of the flagellar structure. It encodes a protein that constitutes the flagellar basal body or hook sheath [54]. Additionally, FleQ activates the *fliA* gene, which encodes the σ28 factor (sigma28, FliA, RpoF) [48,55].

The third-level genes are activated by FliA, leading to the assembly of flagellar filaments, chemotactic signal transduction systems, and the enhancement of the stator complex [56]. For example, *fliC* encodes flagellin (FliC), which, in addition to its motor function, can also adhere to the surface and participate in biofilm formation [57]. As shown in Figure 3A, most flagella are composed of FliC protein.

The bacterial secretion system involves transmembrane transport and protein binding, and the regulation of this system involves genes such as *secY* and *livK*. The *secY* gene encodes the integral membrane protein SecY, which is required by all bacteria to promote protein transmembrane transport. The main mechanism is the generally conserved *secY*/*secE* pathway [58,59]. On the other hand, the *livK* gene encodes an endoplasmic leucine-binding protein LivK, which belongs to the LIV system. This system is responsible for transporting branched-chain amino acids, such as leucine, isoleucine, and valine, into the cytoplasm in an ATP-dependent manner [60].

The adhesion properties of *Pseudomonas* are significantly regulated by NaCl. In *Pseudomonas putida* LP-3, NaCl primarily regulates the *flgC* and *fliC* genes related to flagellar synthesis, resulting in a decrease in flagellar production. This outcome is consistent with the morphological changes that were observed via electron microscopy and leads to a reduced adhesion ability. Simultaneously, NaCl regulation leads to a significant increase in the expression of the *cheB* gene, which is presumed to be an emergency response after the decrease in flagella and motility.

Regarding *Pseudomonas fluorescens* PS01, NaCl mainly regulates the *yajC*, *flgC*, *flgG*, *flgH*, *livK*, and *secY* genes. Among these genes, *flgC*, *flgG*, and *flgH* can influence flagellar production, while *yajC*, *livK*, and *secY* can impact the bacterial secretion system. SecY is highly expressed in strains with strong adhesion and can positively regulate the adhesion of *Pseudomonas*. Adhesion necessitates the support of transmembrane transport [61,62,63,64]. When adhesion occurs, some adhesion proteins must be secreted from the cell to the extracellular space, such as the LivK protein located in the bacterial periplasm. This leads to increased expression of transmembrane transporters [65,66]. The flagella of *Pseudomonas fluorescens* PS01 become more curved before and after regulation, which may be due to the impact of Na^+^ on the flagella dynamic system [67,68,69]. Meanwhile, the surrounding environment becomes turbid, and it is speculated that NaCl regulation can alter the bacterial secretion system-related pathways of *Pseudomonas fluorescens*, thereby modulating the adhesion process of *Pseudomonas fluorescens*. This is vastly different from the NaCl regulation on *Pseudomonas putida*. Although the expression levels of many genes changed after the regulation of *Pseudomonas fluorescens* PS01, the spoilage effect on fish was not as pronounced as expected, potentially due to the inability to provide continuous Na^+^ supply.

Through transcriptome analysis, we postulate that *Pseudomonas* accomplishes flagellar assembly via the regulation of transcription factors, such as RpoN, FleQ, and FliA. As *Pseudomonas* approaches the host surface, specific recognition proteins on the host surface elicit the production of recognition signals, triggering the response of the two-component system and the quorum sensing system. This, in turn, initiates chemotaxis to regulate the flagellar swimming mode and rotation direction, allowing the cell to approach the surface. Simultaneously, specific binding proteins are synthesized within the cell and reach the extracellular space via the cell secretion system. These proteins bind to host proteins to form adhesion.

Furthermore, Na^+^ as an external environmental factor affects the rotation of the flagellar rotor and distorts the flagella, and it can also change the extracellular environment by affecting the secretion system.

## 5. Conclusions

In conclusion, this study investigated the adhesion mechanism of various *Pseudomonas* species based on and identified key findings. LP-3 demonstrated high adhesion ability, while PS01 showed increased adhesion ability after 3.5% NaCl regulation. The increased adhesion of these strains led to an increase in *Nocardioides*, *Cloacibacterium*, and other bacteria in the intestine, as well as the production of volatile compounds, including 2-octen-1-ol Z, 2,3-Octanedione, and Eicosane, which contribute to the spoilage of tilapia. Transcriptomics analysis revealed key adhesion genes, such as *flgG*, *fliC*, and *fliA* genes related to flagellar assembly regulation, and *livK* and *secY* genes associated with the bacterial secretion system. NaCl was found to modulate the expression of these key adhesion genes, thereby affecting the adhesion and spoilage ability of *Pseudomonas*. This study fills the research gap linking the adhesion mechanism to fish spoilage potential by identifying key adhesion genes of two strains of *Pseudomonas* spp. and investigating the effects of NaCl on the adhesion capacity of *Pseudomonas* spp. which provides a theoretical basis for understanding the spoilage mechanisms of *Pseudomonas* spp. Future research can further explore the expression patterns of these key genes under different environmental conditions, as well as develop vaccines or antimicrobial agents based on key adhesion molecules.

## Figures and Tables

**Table 1:** Transcriptome information of adhesion key genes.

Gene Name	Gene Description	Regulation Capacity
*yajC*	preprotein translocase subunit YajC	LP-3>PS01
*flgC*	flagellar basal body rod protein FlgC	LP-3>PS01
*flgG*	flagellar basal body rod protein FlgG	LP-3>PS01
*flgH*	flagellar basal body L-ring protein FlgH	LP-3>PS01
*flgI*	flagellar basal body P-ring protein FlgI	LP-3>PS01
*fliC*	flagellin	LP-3>PS01
*fliA*	RNA polymerase sigma factor FliA	LP-3>PS01
*livK*	branched-chain amino acid ABC transporter substrate-binding protein	LP-3>PS01
*cheB*	chemotaxis response regulator protein-glutamate methylesterase	LP-3>PS01
*secY*	preprotein translocase subunit SecY	LP-3>PS01

## Data Availability

The original contributions presented in this study are included in the article. Further inquiries can be directed to the corresponding authors.
